# Pyridine-3-carboxamidinium chloride

**DOI:** 10.1107/S1600536811002704

**Published:** 2011-03-05

**Authors:** Fei Liu, Fang Zhang, Qifan Chen, Huidong Zhang

**Affiliations:** a College of Chemical Engineering & Materials, Eastern Liaoning University, No. 325 Wenhua Road, Yuanbao District, Dandong City, Liaoning Province 118003, People’s Republic of China; b Experiment Center, Eastern Liaoning University, No. 325 Wenhua Road, Yuanbao District, Dandong City, Liaoning Province 118003, People’s Republic of China

## Abstract

The title compound, C_6_H_8_N_3_^+^·Cl^−^, crystallizes with two formula units in the asymmetric unit. The cations are non-planar with the –C(NH_2_)_2_ groups twisted relative to the ring planes by 36.7 (3) and 37.8 (3)°. The cations are linked into chains through N—H⋯N hydrogen bonds. N—H⋯Cl hydrogen bonds link the chains into a three-dimensional network.

## Related literature

For structures of pyridine-carboxamidinium chlorides, see: Fan *et al.* (2009 ▶); Chen *et al.* (2010 ▶).
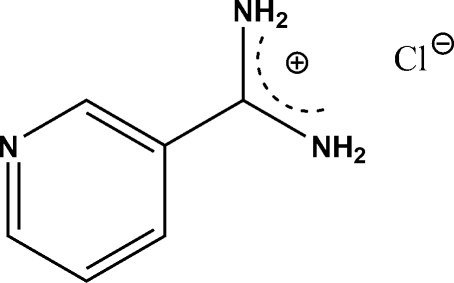


## Experimental

### 

#### Crystal data

C_6_H_8_N_3_^+^·Cl^−^
*M* *_r_* = 157.60
Orthorhombic, 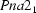

*a* = 10.9485 (7) Å
*b* = 33.1581 (14) Å
*c* = 4.1488 (5) Å
*V* = 1506.1 (2) Å^3^
*Z* = 8
Mo *K*α radiation
μ = 0.43 mm^−1^
*T* = 293 K
0.40 × 0.35 × 0.17 mm

#### Data collection

Rigaku R-AXIS RAPID diffractometer
Absorption correction: multi-scan (*ABSCOR*; Higashi, 1995 ▶) *T*_min_ = 0.845, *T*_max_ = 0.930
14201 measured reflections
3213 independent reflections
2953 reflections with *I* > 2σ(*I*)
*R*_int_ = 0.033

#### Refinement

*R*[*F* ^2^ > 2σ(*F* ^2^)] = 0.029
*wR*(*F* ^2^) = 0.071
*S* = 1.00
3213 reflections
182 parameters
1 restraint
H-atom parameters constrained
Δρ_max_ = 0.18 e Å^−3^
Δρ_min_ = −0.18 e Å^−3^
Absolute structure: Flack (1983 ▶), 1246 Friedel pairs
Flack parameter: 0.0019 (5)

### 

Data collection: *PROCESS-AUTO* (Rigaku, 2006 ▶); cell refinement: *PROCESS-AUTO*; data reduction: *CrystalStructure* (Rigaku/MSC, 2007 ▶); program(s) used to solve structure: *SHELXS97* (Sheldrick, 2008 ▶); program(s) used to refine structure: *SHELXL97* (Sheldrick, 2008 ▶); molecular graphics: *ORTEP-3 for Windows* (Farrugia, 1997 ▶); software used to prepare material for publication: *WinGX* (Farrugia, 1999 ▶).

## Supplementary Material

Crystal structure: contains datablocks global, I. DOI: 10.1107/S1600536811002704/gk2326sup1.cif

Structure factors: contains datablocks I. DOI: 10.1107/S1600536811002704/gk2326Isup2.hkl

Additional supplementary materials:  crystallographic information; 3D view; checkCIF report

## Figures and Tables

**Table 1:** Hydrogen-bond geometry (Å, °)

*D*—H⋯*A*	*D*—H	H⋯*A*	*D*⋯*A*	*D*—H⋯*A*
N3—H3*B*⋯N4	0.86	2.07	2.880 (2)	157
N5—H5*B*⋯Cl2	0.86	2.29	3.1403 (15)	170
N6—H6*B*⋯Cl1	0.86	2.36	3.1562 (16)	155
N2—H2*A*⋯N1^i^	0.86	2.22	2.990 (2)	149
N2—H2*B*⋯Cl2^ii^	0.86	2.31	3.1452 (13)	165
N3—H3*A*⋯Cl2^iii^	0.86	2.27	3.1040 (16)	164
N5—H5*A*⋯Cl1^iv^	0.86	2.46	3.2373 (16)	150
N6—H6*A*⋯Cl1^iv^	0.86	2.41	3.2013 (16)	152

Symmetry codes: (i) 
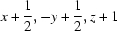
; (ii) 
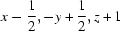
; (iii) 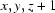; (iv) 
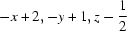
.

**Fig. 1.**
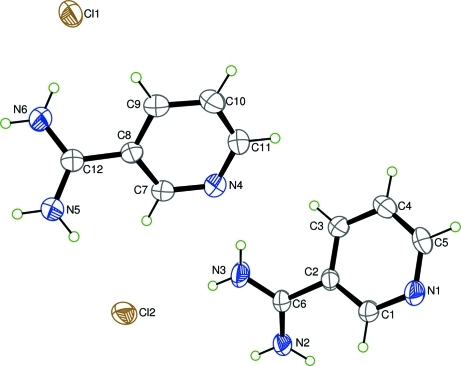
Structure of the title compound showing 30% probability displacement ellipsoids.

**Fig. 2.**
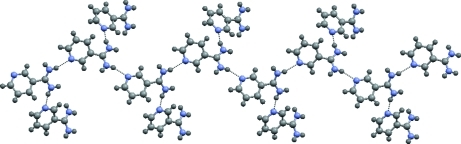
View of one-dimensional supramolecular chain in the title compound.

**Fig. 3.**
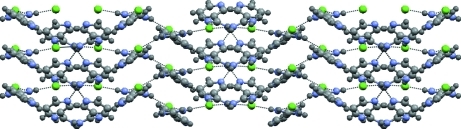
The crystal packing of the title compound shown down the x axis. Hydrogen bonds are shown with dashed lines.

## supplementary crystallographic information

### Comment

During the last decade, much interest has been focused on the synthesis of pyridine carboximidamidate derivatives because of their very potent antibacterial activities, interesting biological properties and applications in coordination chemistry.

The title compound is an organic salt which crystallizes with two formula units in the asymmetric unit (Fig. 1). The molecules are nonplanar with the C(NH_2_)_2_ groups twisted out of the ring planes with the twist angles of 36.7 (3) ° and 37.8 (3) °. The bond lengths and angles in title compound are in the normal ranges comparable with those in the related structure (Fan *et al.*, 2009).

In the crystal structure, pyridine-3-carboximidamidate cations are linked by N—H···N hydrogen bonds to form one-dimensional supramolecular molecular chain (Fig. 2). Cl1 ion bridges two pyridine carboximidamidate cations through N—H···Cl hydrogen bonding and Cl2 ion triple-bridges three cations through N—H···Cl hydrogen bonding. Thus, one-dimensional chains of cations are linked by N—H···Cl interactions into a three-dimensional network (Fig. 3).

### Experimental

To a solution of sodium methoxide (5.15 mmol) in methnol (50 ml) was added 3-cyanopyridine (5.2 g, 4.99 mmol). The mixture was stirred at room temperature for 2 h. Then ammonium chloride (2.9 g, 5.42 mmol) was slowly added to the resulting solution and the mixture was stirred at room temperature for 48 h. The resulting suspension was filtered and the solvent was removed from the filtrate under reduced pressure. Purification by washing with diethylether gave 3-amidinopyridine chloride (5.74 g, 94%) as an off-white solid. Block-shaped crystals suitable for X-ray diffraction were obtained by recrystallization from from ethanol.

### Refinement

H atoms were placed in calculated positions and treated as riding on their parent atoms (C—H = 0.93 Å, N-H = 0.86 Å) and *U*_iso_(H) = 1.2U_eq_(C,N).

### Crystal data

**Table:**

C_6_H_8_N_3_^+^·Cl^−^	*F*(000) = 656
*M**_r_* = 157.60	*D*_x_ = 1.390 Mg m^−^^3^
Orthorhombic, *P**n**a*2_1_	Mo *K*α radiation, λ = 0.71073 Å
Hall symbol: P 2c -2n	Cell parameters from 3213 reflections
*a* = 10.9485 (7) Å	θ = 3.1–27.5°
*b* = 33.1581 (14) Å	µ = 0.43 mm^−^^1^
*c* = 4.1488 (5) Å	*T* = 293 K
*V* = 1506.1 (2) Å^3^	Block, colorless
*Z* = 8	0.40 × 0.35 × 0.17 mm

### Data collection

**Table:**

Rigaku R-AXIS RAPID diffractometer	3213 independent reflections
Radiation source: fine-focus sealed tube	2953 reflections with *I* > 2σ(*I*)
graphite	*R*_int_ = 0.033
Detector resolution: 10.0 pixels mm^-1^	θ_max_ = 27.5°, θ_min_ = 3.1°
ω scans	*h* = −14→14
Absorption correction: multi-scan (*ABSCOR*; Higashi, 1995)	*k* = −42→42
*T*_min_ = 0.845, *T*_max_ = 0.930	*l* = −4→5
14201 measured reflections	

### Refinement

**Table:**

Refinement on *F*^2^	Hydrogen site location: inferred from neighbouring sites
Least-squares matrix: full	H-atom parameters constrained
*R*[*F*^2^ > 2σ(*F*^2^)] = 0.029	*w* = 1/[σ^2^(*F*_o_^2^) + (0.0334*P*)^2^ + 0.3352*P*] where *P* = (*F*_o_^2^ + 2*F*_c_^2^)/3
*wR*(*F*^2^) = 0.071	(Δ/σ)_max_ = 0.002
*S* = 1.00	Δρ_max_ = 0.18 e Å^−^^3^
3213 reflections	Δρ_min_ = −0.17 e Å^−^^3^
182 parameters	Extinction correction: *SHELXL97* (Sheldrick, 2008), Fc^*^=kFc[1+0.001xFc^2^λ^3^/sin(2θ)]^-1/4^
1 restraint	Extinction coefficient: 0.061 (2)
Primary atom site location: structure-invariant direct methods	Absolute structure: Flack (1983), 1246 Friedel pairs
Secondary atom site location: difference Fourier map	Flack parameter: 0.0019 (5)

### Special details

**Table:**

Geometry. All e.s.d.'s (except the e.s.d. in the dihedral angle between two l.s. planes) are estimated using the full covariance matrix. The cell e.s.d.'s are taken into account individually in the estimation of e.s.d.'s in distances, angles and torsion angles; correlations between e.s.d.'s in cell parameters are only used when they are defined by crystal symmetry. An approximate (isotropic) treatment of cell e.s.d.'s is used for estimating e.s.d.'s involving l.s. planes.
Refinement. Refinement of *F*^2^ against ALL reflections. The weighted *R*-factor *wR* and goodness of fit *S* are based on *F*^2^, conventional *R*-factors *R* are based on *F*, with *F* set to zero for negative *F*^2^. The threshold expression of *F*^2^ > σ(*F*^2^) is used only for calculating *R*-factors(gt) *etc*. and is not relevant to the choice of reflections for refinement. *R*-factors based on *F*^2^ are statistically about twice as large as those based on *F*, and *R*- factors based on ALL data will be even larger.

### Fractional atomic coordinates and isotropic or equivalent isotropic displacement parameters (Å^2^)

**Table:**

	*x*	*y*	*z*	*U*_iso_*/*U*_eq_	
Cl1	0.80408 (4)	0.549620 (13)	0.75756 (15)	0.05193 (15)	
Cl2	0.82982 (4)	0.318730 (12)	0.22533 (15)	0.04986 (15)	
N2	0.49341 (12)	0.25905 (4)	1.1812 (4)	0.0384 (3)	
H2A	0.5635	0.2541	1.2661	0.046*	
H2B	0.4372	0.2409	1.1835	0.046*	
N1	0.17495 (13)	0.28055 (4)	0.6167 (4)	0.0392 (4)	
N4	0.56768 (13)	0.39520 (4)	0.6649 (4)	0.0427 (4)	
C3	0.30154 (15)	0.34172 (5)	0.9320 (5)	0.0359 (4)	
H3	0.3444	0.3622	1.0348	0.043*	
C2	0.35090 (13)	0.30307 (5)	0.9096 (4)	0.0294 (3)	
N3	0.55529 (14)	0.32232 (5)	1.0408 (6)	0.0540 (5)	
H3A	0.6261	0.3181	1.1239	0.065*	
H3B	0.5390	0.3451	0.9521	0.065*	
C1	0.28379 (13)	0.27351 (4)	0.7498 (5)	0.0321 (3)	
H1	0.3164	0.2477	0.7352	0.038*	
N5	0.93657 (14)	0.40511 (4)	0.3554 (5)	0.0515 (5)	
H5A	1.0143	0.4075	0.3329	0.062*	
H5B	0.9030	0.3817	0.3431	0.062*	
N6	0.91881 (14)	0.47268 (4)	0.4274 (5)	0.0511 (5)	
H6A	0.9965	0.4754	0.4053	0.061*	
H6B	0.8739	0.4935	0.4622	0.061*	
C7	0.68741 (16)	0.40022 (5)	0.6164 (5)	0.0376 (4)	
H7	0.7409	0.3809	0.6971	0.045*	
C12	0.86919 (16)	0.43713 (5)	0.4083 (5)	0.0366 (4)	
C6	0.47248 (14)	0.29402 (5)	1.0493 (5)	0.0328 (3)	
C5	0.12821 (16)	0.31783 (5)	0.6479 (5)	0.0448 (5)	
H5	0.0512	0.3228	0.5624	0.054*	
C8	0.73540 (15)	0.43301 (4)	0.4505 (4)	0.0332 (4)	
C4	0.18844 (16)	0.34905 (5)	0.7998 (5)	0.0440 (5)	
H4	0.1533	0.3745	0.8126	0.053*	
C9	0.65596 (16)	0.46198 (5)	0.3279 (5)	0.0400 (4)	
H9	0.6853	0.4846	0.2198	0.048*	
C11	0.49241 (17)	0.42297 (5)	0.5413 (5)	0.0432 (4)	
H11	0.4089	0.4196	0.5718	0.052*	
C10	0.53172 (17)	0.45609 (5)	0.3720 (5)	0.0433 (4)	
H10	0.4757	0.4744	0.2879	0.052*	

### Atomic displacement parameters (Å^2^)

**Table:**

	*U*^11^	*U*^22^	*U*^33^	*U*^12^	*U*^13^	*U*^23^
Cl1	0.0348 (2)	0.0464 (2)	0.0746 (4)	0.00362 (16)	0.0023 (3)	−0.0112 (3)
Cl2	0.0345 (2)	0.03388 (19)	0.0812 (4)	0.00596 (15)	−0.0204 (2)	−0.0130 (2)
N2	0.0260 (6)	0.0361 (7)	0.0531 (10)	−0.0005 (5)	−0.0083 (6)	0.0086 (7)
N1	0.0318 (7)	0.0404 (7)	0.0454 (9)	−0.0045 (6)	−0.0117 (6)	0.0005 (7)
N4	0.0342 (7)	0.0342 (6)	0.0597 (11)	−0.0032 (5)	0.0011 (7)	0.0042 (7)
C3	0.0339 (8)	0.0309 (8)	0.0428 (10)	−0.0015 (6)	−0.0054 (7)	−0.0025 (8)
C2	0.0230 (7)	0.0320 (7)	0.0332 (8)	−0.0020 (6)	−0.0022 (6)	0.0043 (7)
N3	0.0291 (7)	0.0421 (8)	0.0907 (14)	−0.0092 (6)	−0.0213 (8)	0.0190 (9)
C1	0.0292 (7)	0.0307 (7)	0.0363 (9)	−0.0015 (5)	−0.0041 (8)	0.0015 (8)
N5	0.0326 (7)	0.0328 (7)	0.0890 (15)	0.0001 (6)	0.0051 (8)	−0.0079 (8)
N6	0.0359 (8)	0.0336 (7)	0.0837 (14)	−0.0037 (6)	0.0103 (8)	−0.0099 (9)
C7	0.0348 (8)	0.0285 (7)	0.0495 (11)	0.0022 (6)	−0.0024 (7)	0.0008 (8)
C12	0.0347 (8)	0.0318 (7)	0.0433 (10)	0.0002 (6)	0.0015 (7)	−0.0025 (8)
C6	0.0239 (7)	0.0348 (8)	0.0396 (9)	−0.0021 (6)	−0.0046 (7)	0.0004 (7)
C5	0.0281 (8)	0.0510 (10)	0.0552 (13)	0.0046 (7)	−0.0141 (8)	0.0008 (9)
C8	0.0324 (8)	0.0276 (7)	0.0397 (10)	−0.0003 (6)	−0.0010 (7)	−0.0046 (7)
C4	0.0364 (8)	0.0391 (8)	0.0565 (13)	0.0098 (7)	−0.0067 (8)	−0.0020 (9)
C9	0.0413 (9)	0.0313 (7)	0.0473 (12)	0.0021 (7)	−0.0011 (8)	0.0036 (7)
C11	0.0306 (8)	0.0410 (9)	0.0580 (12)	−0.0010 (7)	−0.0038 (8)	−0.0026 (9)
C10	0.0400 (9)	0.0383 (9)	0.0516 (11)	0.0076 (7)	−0.0066 (8)	0.0031 (8)

### Geometric parameters (Å, °)

**Table:**

N2—C6	1.303 (2)	N5—H5A	0.8600
N2—H2A	0.8600	N5—H5B	0.8600
N2—H2B	0.8600	N6—C12	1.300 (2)
N1—C1	1.334 (2)	N6—H6A	0.8600
N1—C5	1.344 (2)	N6—H6B	0.8600
N4—C7	1.337 (2)	C7—C8	1.390 (2)
N4—C11	1.338 (2)	C7—H7	0.9300
C3—C4	1.376 (2)	C12—C8	1.482 (2)
C3—C2	1.394 (2)	C5—C4	1.380 (3)
C3—H3	0.9300	C5—H5	0.9300
C2—C1	1.393 (2)	C8—C9	1.392 (2)
C2—C6	1.483 (2)	C4—H4	0.9300
N3—C6	1.305 (2)	C9—C10	1.386 (3)
N3—H3A	0.8600	C9—H9	0.9300
N3—H3B	0.8600	C11—C10	1.373 (3)
C1—H1	0.9300	C11—H11	0.9300
N5—C12	1.311 (2)	C10—H10	0.9300
			
C6—N2—H2A	120.0	C8—C7—H7	118.6
C6—N2—H2B	120.0	N6—C12—N5	120.60 (16)
H2A—N2—H2B	120.0	N6—C12—C8	119.31 (15)
C1—N1—C5	117.47 (14)	N5—C12—C8	120.08 (14)
C7—N4—C11	117.45 (16)	N2—C6—N3	121.94 (15)
C4—C3—C2	118.99 (16)	N2—C6—C2	120.16 (14)
C4—C3—H3	120.5	N3—C6—C2	117.88 (15)
C2—C3—H3	120.5	N1—C5—C4	123.50 (16)
C1—C2—C3	118.31 (14)	N1—C5—H5	118.2
C1—C2—C6	121.15 (14)	C4—C5—H5	118.2
C3—C2—C6	120.54 (14)	C7—C8—C9	118.99 (16)
C6—N3—H3A	120.0	C7—C8—C12	120.30 (15)
C6—N3—H3B	120.0	C9—C8—C12	120.71 (15)
H3A—N3—H3B	120.0	C3—C4—C5	118.66 (15)
N1—C1—C2	123.03 (14)	C3—C4—H4	120.7
N1—C1—H1	118.5	C5—C4—H4	120.7
C2—C1—H1	118.5	C10—C9—C8	117.87 (16)
C12—N5—H5A	120.0	C10—C9—H9	121.1
C12—N5—H5B	120.0	C8—C9—H9	121.1
H5A—N5—H5B	120.0	N4—C11—C10	123.61 (17)
C12—N6—H6A	120.0	N4—C11—H11	118.2
C12—N6—H6B	120.0	C10—C11—H11	118.2
H6A—N6—H6B	120.0	C11—C10—C9	119.20 (17)
N4—C7—C8	122.86 (16)	C11—C10—H10	120.4
N4—C7—H7	118.6	C9—C10—H10	120.4
			
C4—C3—C2—C1	0.8 (3)	N4—C7—C8—C12	179.21 (18)
C4—C3—C2—C6	−179.98 (18)	N6—C12—C8—C7	−141.6 (2)
C5—N1—C1—C2	−1.7 (3)	N5—C12—C8—C7	37.8 (3)
C3—C2—C1—N1	0.2 (3)	N6—C12—C8—C9	37.8 (3)
C6—C2—C1—N1	−178.99 (18)	N5—C12—C8—C9	−142.8 (2)
C11—N4—C7—C8	1.2 (3)	C2—C3—C4—C5	−0.4 (3)
C1—C2—C6—N2	−36.7 (3)	N1—C5—C4—C3	−1.2 (3)
C3—C2—C6—N2	144.10 (19)	C7—C8—C9—C10	−1.4 (3)
C1—C2—C6—N3	144.72 (19)	C12—C8—C9—C10	179.20 (17)
C3—C2—C6—N3	−34.5 (3)	C7—N4—C11—C10	−0.6 (3)
C1—N1—C5—C4	2.1 (3)	N4—C11—C10—C9	−1.0 (3)
N4—C7—C8—C9	−0.2 (3)	C8—C9—C10—C11	1.9 (3)

### Hydrogen-bond geometry (Å, °)

**Table:**

*D*—H···*A*	*D*—H	H···*A*	*D*···*A*	*D*—H···*A*
N3—H3B···N4	0.86	2.07	2.880 (2)	157
N5—H5B···Cl2	0.86	2.29	3.1403 (15)	170
N6—H6B···Cl1	0.86	2.36	3.1562 (16)	155
N2—H2A···N1^i^	0.86	2.22	2.990 (2)	149
N2—H2B···Cl2^ii^	0.86	2.31	3.1452 (13)	165
N3—H3A···Cl2^iii^	0.86	2.27	3.1040 (16)	164
N5—H5A···Cl1^iv^	0.86	2.46	3.2373 (16)	150
N6—H6A···Cl1^iv^	0.86	2.41	3.2013 (16)	152

Symmetry codes: (i) *x*+1/2, −*y*+1/2, *z*+1; (ii) *x*−1/2, −*y*+1/2, *z*+1; (iii) *x*, *y*, *z*+1; (iv) −*x*+2, −*y*+1, *z*−1/2.
